# Primary Multidrug Resistant Tuberculosis and Utility of Line Probe Assay for Its Detection in Smear-Positive Sputum Samples in a Tertiary Care Hospital in South India

**DOI:** 10.1155/2016/6235618

**Published:** 2016-03-23

**Authors:** Fahmiya Leena Yacoob, Beena Philomina Jose, Sarada Devi Karunakaran Lelitha, Sreelatha Sreenivasan

**Affiliations:** Department of Microbiology, Government Medical College, Kozhikode, Kerala 673008, India

## Abstract

In a high tuberculosis burdened country like India, rapid, cost-effective, and reliable diagnostic tools for tuberculosis are an urgent need of the hour to prevent inappropriate treatment strategies and further spread of resistance. This study aimed to estimate the proportion of new smear-positive tuberculosis cases with primary resistance to rifampicin and/or isoniazid as well as identify the common mutations associated with it. Sputum of 200 newly diagnosed smear-positive cases of 1+ score and above was directly subjected to Line Probe Assay using the GenoType MTBDR*plus* assay kit. All samples were inoculated onto solid media and 61 samples were inoculated in automated liquid culture also. The Line Probe Assay gave hundred percent interpretable results with 2.5% of the study population showing resistant pattern. Only 1% of the cases were primary multidrug resistant tuberculosis and 1.5% showed isoniazid monoresistance. S531L and C15T were the most common genetic mutations seen for rifampicin and isoniazid resistance, respectively. 40% had absent* rpoB* wild type 8 band indicating probable silent mutation after clinical correlation. The average turnaround time for Line Probe Assay was far less (3.8 days) as compared to solid and liquid cultures (35.6 days and 13.5 days, resp.).

## 1. Introduction

Tuberculosis (TB) earned the sobriquet “The Captain of All These Men of Death” during the 18th and 19th century for the morbidity and mortality it caused and now ranks alongside HIV as a leading cause of death worldwide [1]. India ranks first among the high TB burdened countries contributing to 24% of the estimated global incidence. It is also one of the three countries contributing to more than half of the global burden of multidrug resistant tuberculosis (MDR TB), the other two being China and the Russian Federation [2, 3].

Improved rapid diagnostic methods, vigilant monitoring of case reporting, and follow-up through national surveillance programs in these countries will have a global impact on the TB statistics. Although MDR TB is reported to be low with 2.2% among new cases and 15% among retreatment cases, due to the large population and high TB burden, it translates into a high number of actual cases (71,000 estimated MDR TB cases among notified pulmonary TB cases in 2014) [1]. Sputum smear microscopy remains the mainstay of diagnosis due to its simplicity, cost-effectiveness, and feasibility. However, molecular diagnostic techniques like GeneXpert, Polymerase Chain Reaction (PCR), and Line Probe Assays (LPAs) are being increasingly used recently. These newer techniques aid in the rapid diagnosis of drug resistance and thus prevent inappropriate treatment regimes, amplification, and spread of drug resistance in countries like India with high TB load. The World Health Organization (WHO) recommended a new policy to use LPAs for rapid screening of patients at risk of MDR TB in 2008 [4].

The LPA is a reverse hybridization technique wherein the patient's sample, after undergoing DNA extraction and amplification, is subjected to hybridization with membrane strips coated with complementary probes targeted against specific genes. Highly specific binding of complementary DNA strands is ensured by stringent conditions which result from the combination of buffer composition and a certain temperature. During hybridization, streptavidin-conjugated alkaline phosphatase is added which binds to the amplicon's biotin via the streptavidin moiety. Finally, the alkaline phosphatase transforms an added substrate into a dye which become visible on the membrane strips as a coloured precipitate. A template helps in the interpretation of the banding patterns obtained.

GenoType MTBDR*plus* assay (Hain Lifesciences, GmbH, Nehren, Germany) targets the* rpoB* (coding for beta subunit of RNA polymerase),* katG* (coding for catalase peroxidase), and promoter region of* inhA* (coding for NADH enoyl ACP reductase) genes in both culture isolates and clinical samples. Thus, in addition to rifampicin resistance (*rpoB* gene), MTBDR*plus* assay aids in the detection of high level and low level resistance to isoniazid (INH) via the* katG* gene and* inhA* gene, respectively. In India, as per the Revised National TB Control Programme (RNTCP), the definition of presumptive drug resistant TB (DR TB) cases includes all retreatment cases at diagnosis, any smear-positive cases during follow-up, contacts of confirmed DR TB cases, and HIV associated TB cases at diagnosis [5]. For drug susceptibility testing (DST), rapid molecular tests like LPA and CB-NAAT (cartridge based-nucleic acid amplification tests), if available, are the preferred DST methods for first-line drugs as per RNTCP protocol [5]. This study aimed at finding the proportion of cases with primary drug resistance in the study population and to assess the utility of LPA in our set-up in comparison to solid and automated liquid culture systems.

## 2. Materials and Methods

### 2.1. Specimen Collection and Storage

Early morning expectorated sputum samples of 200 new smear-positive cases of grade 1+ and above were collected in sterile leakproof containers during the study period of one and a half years. All the samples were stored at a temperature of 2–8°C for not more than 4 days before decontamination.

### 2.2. Specimen Processing

#### 2.2.1. Direct Smears

The specimens were stained by Ziehl-Neelsen technique. The smears were graded as “scanty” for 1–10 AFB/100 oil emulsion fields (OIF), “1+” for 10–99 AFB/100 OIF, “2+” for 1–9 AFB/OIF, and “3+” for >10 AFB/OIF. Smears graded “scanty” positive were excluded from this study.

#### 2.2.2. Decontamination

It was done by N-acetyl-L-cysteine- (NALC-) NaOH-sodium citrate method in which equal amounts of 4% NaOH and 2.9% sodium citrate were mixed with NALC powder (0.5 g NALC/100 mL of NaOH-sodium citrate solution). This mixture was used within 24 hours of preparation. In a 50 mL plastic sterile conical bottom graduated centrifuge tube, specimen ≤ 10 mL and NALC solution were added in equal volume. The capped tubes were vortexed (about 5–20 seconds/tube) and after allowing it to stand for 15–20 minutes, the tubes were filled to 50 mL with sterile phosphate buffer solution at pH 6.8 and swirled by hand to mix. The specimens were then concentrated in a centrifuge at a speed of 3,000 g for 15 minutes and the supernatant fluid was carefully decanted from the pellet. The pellet sediments were resuspended with phosphate buffer (pH 6.8) to achieve a final volume of 1 mL.

#### 2.2.3. Culture

All samples were subjected to solid culture on Lowenstein-Jensen (LJ) media. Sixty-one samples were inoculated into MB-Bact automated liquid culture media. This protocol was made to ensure that, in cases where sputum samples gave invalid/negative result with direct LPA, their culture isolates can be processed as per the manufacturer's instructions and subjected to LPA (GenoType MTBDR*plus* ver 2.0). However, none of the samples gave invalid results.

#### 2.2.4. DNA Extraction and Amplification

The decontaminated samples were stored at −20°C for not more than 5 days until DNA extraction was carried out. 500 *μ*L of the decontaminated specimen was taken in a 1.5 mL microfuge tube and centrifuged at 10,000 g for 15 minutes. The pellet was resuspended in 200 *μ*L of molecular biology grade water and incubated at 95°C for 20 minutes in a water bath. Then it was incubated for 15 minutes in an ultrasonic bath. It was spun down for 5 minutes at 14,000 g and 5 *μ*L of the supernatant was used directly for PCR.

After DNA extraction, each sample (5 *μ*L) was mixed with 10 *μ*L AM-A and 35 *μ*L AM-B (amplification mix provided with the kit) to make a final volume of 50 *μ*L. Instead of the extracted DNA solution, 5 *μ*L of molecular biology grade water was added to the negative control. Amplification was done in thermal cycler as per the kit manual [6]. In case of delay, the amplification products were stored at −20°C until further use.

#### 2.2.5. Hybridization

The Hain protocol as per the manufacturer's instructions was followed.

Incubation was done in “TwinCubator” which is a semiautomatic washing and shaking device with a capacity to hold 12 samples at a time. Hybridization steps were performed using reagents provided with the kit. 20 *μ*L of Denaturation Solution (DEN) was dispensed in a corner of each of the wells used. The amplified products were then carefully added and mixed. After five minutes of incubation, hybridization buffer was added and a single strip was placed in each well with coated side facing up. Following incubation and washing steps, the diluted conjugate was added and kept for further incubation. Finally, the substrate was added and the strips were incubated protected from light. Washing steps with stringent and rinse wash solutions were performed as required by the kit protocol. After final wash step with distilled water, the strips were removed using tweezers and dried between 2 layers of absorbent paper. The developed sheets were pasted on the evaluation sheet provided with the kit in the designated fields by aligning the bands CC and AC with the respective lines.

Each strip has a total of 27 reaction zones. The presence of wild type bands without a mutant band is interpreted as sensitive and the presence of a mutant band with or without the simultaneous absence of the corresponding wild type band is interpreted as resistant pattern.

All patients were registered under the RNTCP Unit of Government Medical College, Kozhikode, and they were on regular follow-up. All smear-positive patients were initiated on category I regimen of “directly observed treatment short-course” (DOTS) and those with confirmed MDR TB were initiated on category IV regimen (DOTS*plus*) (Table 1).

## 3. Results

**Figure 1:**
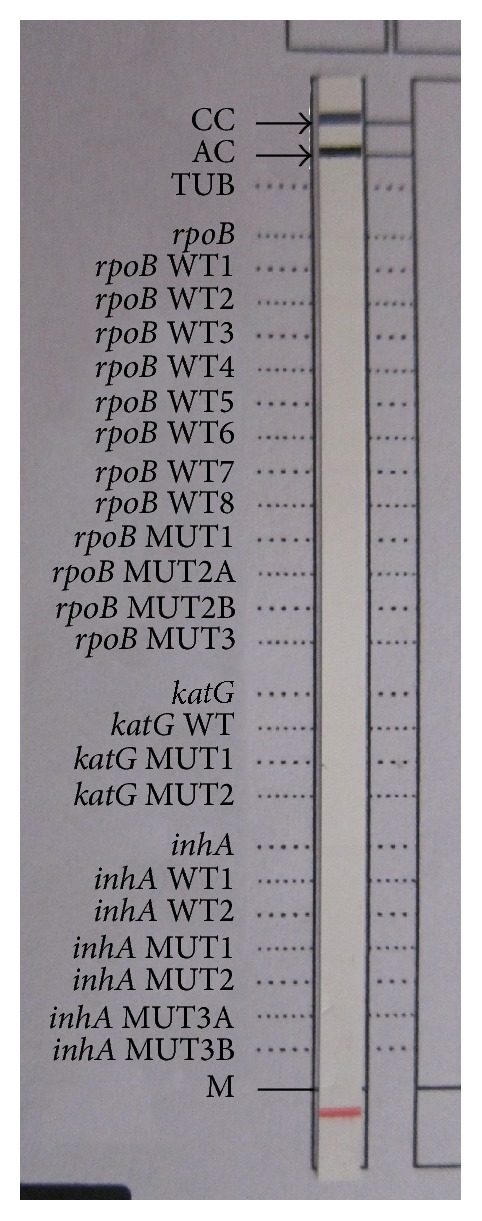
Negative control with CC and AC bands. The red band is reference marker to align the strip.

A total of 200 new sputum smear-positive samples were processed and directly subjected to LPA. All the samples gave valid and interpretable results as indicated by the presence of all control loci bands. The negative controls showed the presence of conjugate control (CC) and amplification control (CC) bands only (Figure 1). All the samples showed presence of TUB band thereby suggesting infection with* Mycobacterium tuberculosis* complex.

Out of the 200 samples, 1% (2 samples) showed MDR TB pattern on LPA. An additional 1.5% (3 samples) showed INH monoresistance in this study.

**Figure 2:**
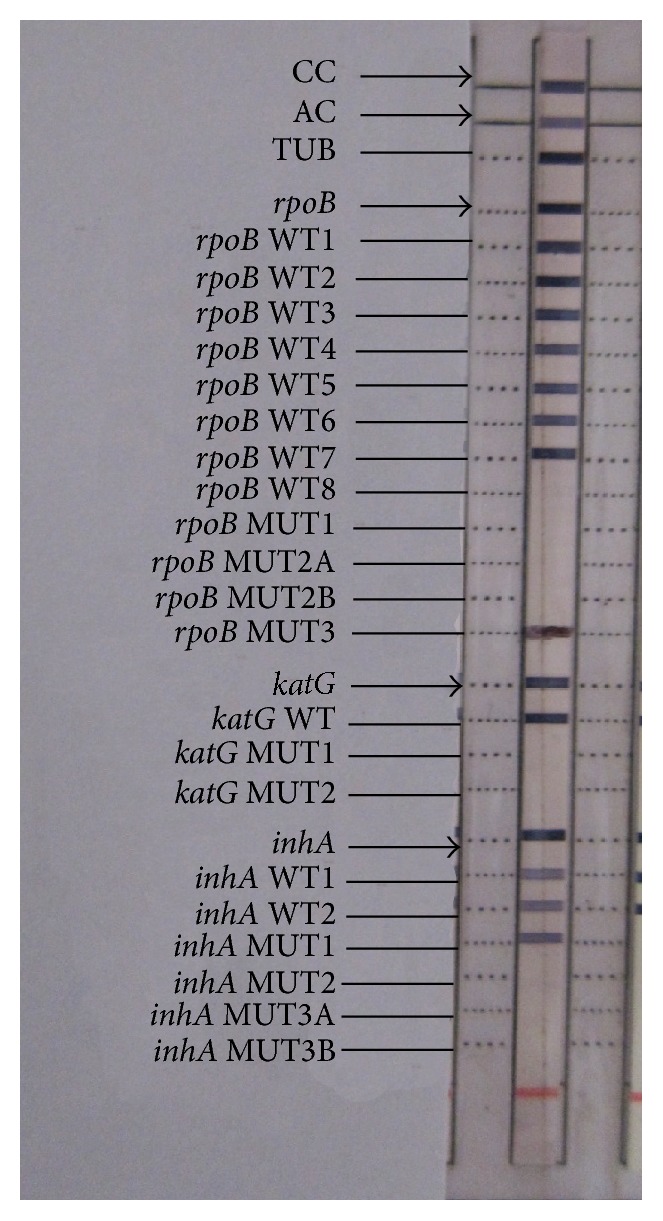
Patient with MDR TB showing presence of* rpoB* MUT3 band and* inhA* MUT1 band along with the wild type bands indicating rifampicin resistance and INH heteroresistance, respectively.

**Figure 3:**
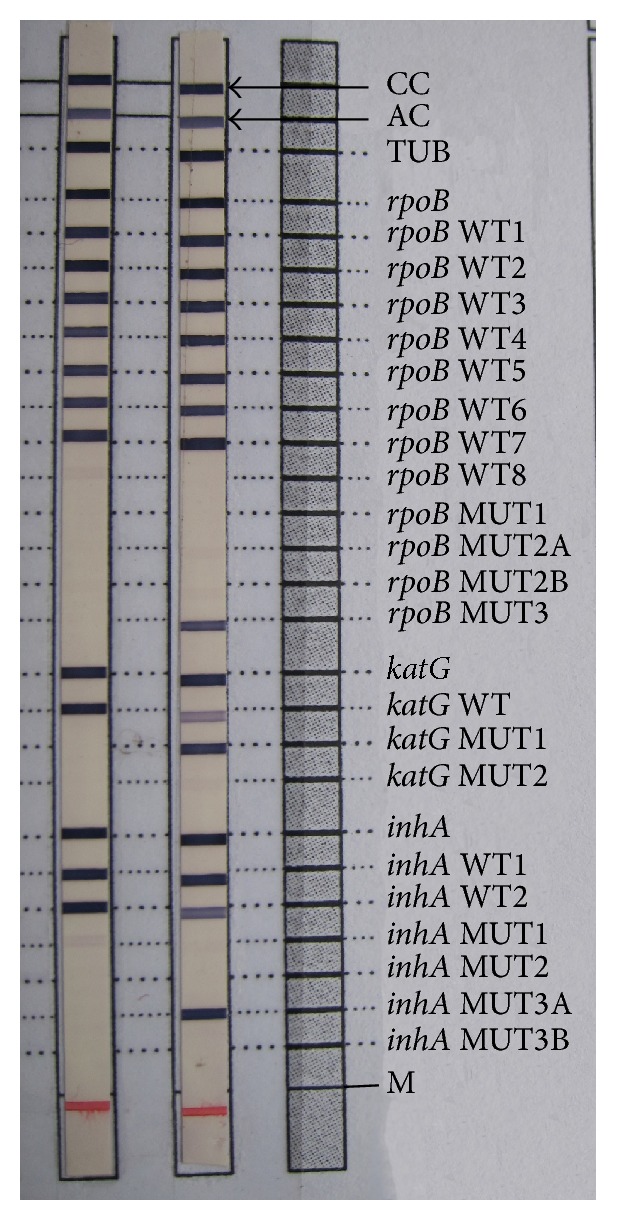
(From left to right) Lane 1: sensitive pattern. Lane 2: MDR TB patient with* rpoB* MUT3 band indicating rifampicin resistance and* katG* MUT1 band and* inhA* MUT3A band along with wild type band indicating INH heteroresistance. Lane 3: reference strip.

The two samples with MDR TB pattern on LPA were confirmed from an Intermediate Reference Laboratory (IRL) in Kerala by both LPA and phenotypic DST. Both the MDR TB isolates in this study showed absent* rpoB* WT8 band and presence of* rpoB* MUT3 band (Figures 2 and 3) which represents a mutation and amino acid change from serine to leucine associated with codon number 531 of the* rpoB* gene.

A significant association was found between the presence of rifampicin resistance and INH resistance in this study as both the samples with rifampicin resistance had resistance to INH also.

Among the two MDR cases detected in this study, one patient had mutation in both the high level and low level INH resistance determining regions indicated by the presence of* katG* MUT1 band corresponding to S315T1 mutation in codon 315 of* katG* gene and* inhA* MUT3A band corresponding to T8C mutation in the -8 nucleic acid position of the* inhA* promoter region, respectively. Both the INH mutations showed heteroresistant pattern; that is, there was simultaneous presence of both mutant and wild type bands for the specified genes.

The other MDR sample showed only low level INH heteroresistance indicated by the presence of* inhA* MUT1 band corresponding to C15T mutation in the -15 nucleic acid position of the* inhA* promoter region.

Both the patients were diabetic and one of them was HIV positive. They were initiated on category IV regimen after confirmation of this study's findings by phenotypic DST.

**Figure 4:**
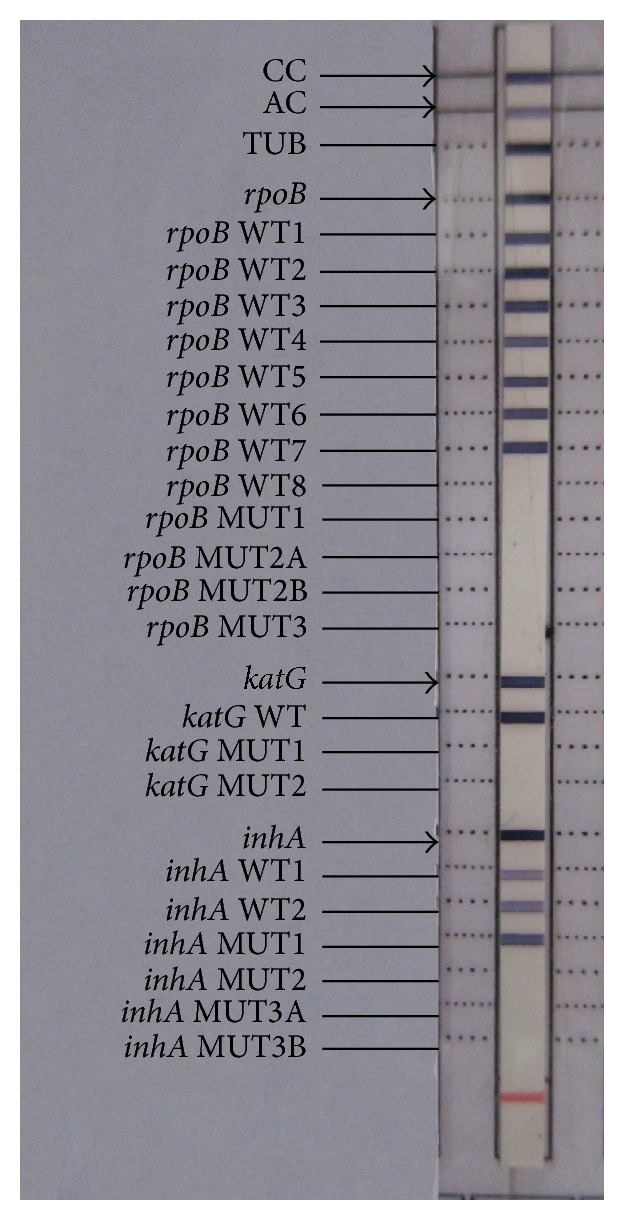
Patient with* inhA* MUT1 band along with wild type bands indicating low level INH heteroresistance.

Out of the 3 patients (1.5%) with INH monoresistance, 2 patients had low level INH heteroresistance showing* inhA* MUT1 band corresponding to C15T mutation in the -15 nucleic acid position of the* inhA* promoter region with simultaneous presence of wild type bands (Figure 4). Both the patients improved under category I treatment regimen.

**Figure 5:**
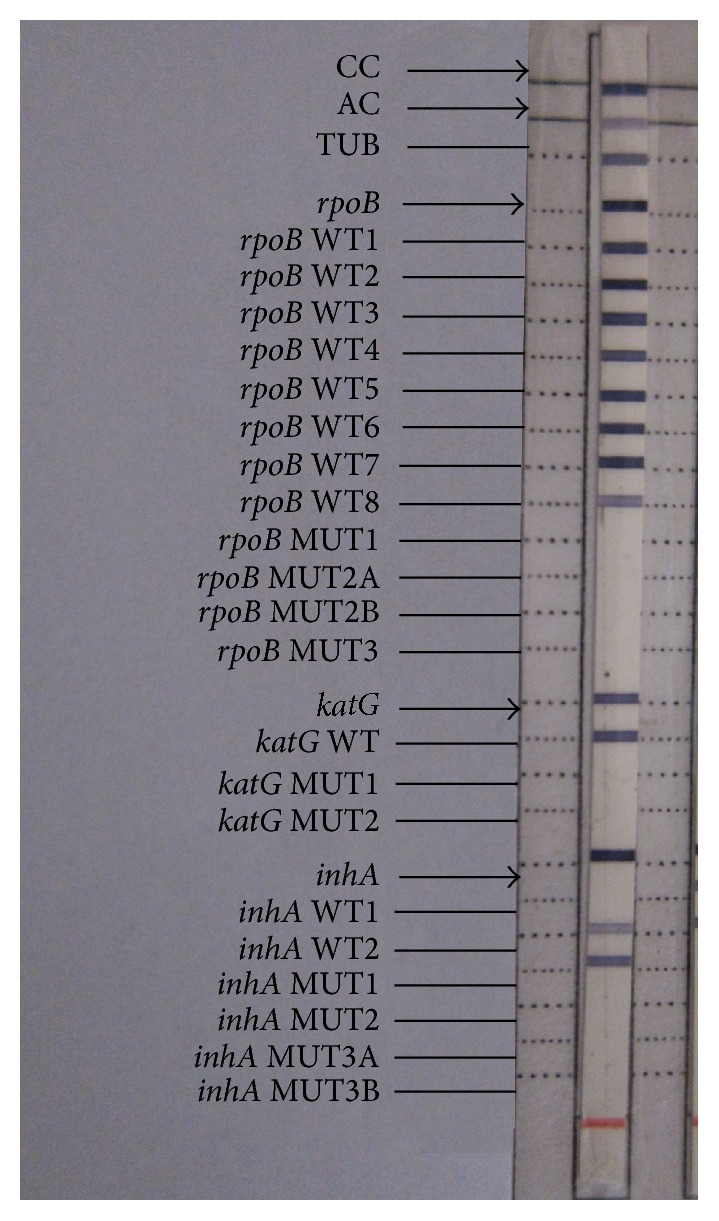
Patient showing low level INH monoresistance with absent* inhA* WT1 band and presence of* inhA* MUT1 band.

The third patient had similar mutation, C15T, indicating low level INH monoresistance but with absent wild type band (Figure 5). This patient had coexisting morbidities of diabetic kidney disease with bilateral hydroureteronephrosis and renal TB. She succumbed to death while on category I treatment. However the cause of the death cannot be completely attributed to INH resistance due to her otherwise poor health condition.

**Figure 6:**
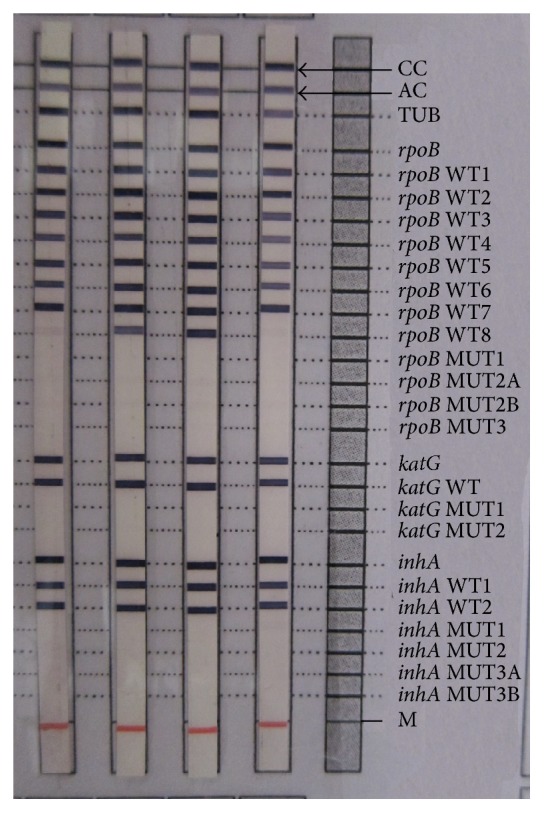
(From left to right) Lane 1: faint* rpoB* WT8 band interpreted as sensitive pattern. Lanes 2 and 3: sensitive pattern. Lane 4: sensitive pattern with absent* rpoB* WT8 band.

Another interesting observation in this study was the absence of* rpoB* WT8 band in almost 40% of the samples (Figure 6). Faint band was taken as positive as per the manual along with the kit.

All the patients with only absent* rpoB* WT8 band responded to category I regimen with follow-up sputum smears being negative.

### 3.1. Comparison of LPA and Solid and Automated Liquid Culture

The isolation rate in LJ medium in the present study was 79% and that in MB-Bact was 91.8%. In comparison, the LPA gave a valid result in all 200 samples.

In the present study, average detection time was 35.6 days by conventional solid culture (Range 17–50 days) and 13.5 days by automated MB-Bact system (Range 5–25 days). On the other hand, the average turnaround time of LPA from receipt of sample to the reporting of result was 3.8 days (Range 2–8 days).

### 3.2. Epidemiological Characteristics of Study Population

Of the 200 samples, 162 (81%) were from males with a female-to-male ratio of 0.23 : 1.

**Figure 7:**
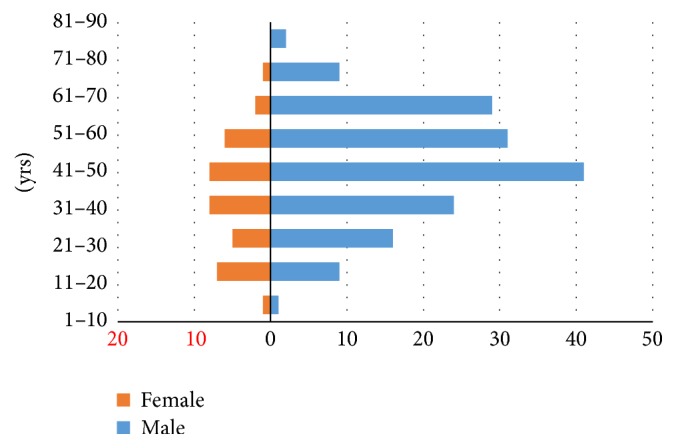
Age and gender distribution of the study population. The majority of the patients are of the age group between 41 and 50 with male preponderance.

The age and sex distribution of the study population is depicted in Figure 7.

In this study, 4.5% of the study population were positive for HIV which is consistent with the RNTCP finding stating that the TB epidemic in India continues to be predominantly driven by the pool of HIV negative TB infected individuals [7].

Among the tuberculosis patients with diabetes, 77.6% had higher smear grades of 2+ and 3+. This study showed a significant association between diabetes mellitus (DM) and smear grade with a higher bacillary load being observed in diabetic patients (Table 2). DM has received recent recognition as a risk factor for TB and epidemiological studies have elucidated an association between DM and the development of TB [8].

## 4. Discussion

This study was conducted in a tertiary care hospital in Northern Kerala, Government Medical College, Kozhikode, to assess the proportion of primary MDR TB among new sputum smear-positive cases and the utility of LPA for its rapid detection. This study also aimed to find the most common genetic mutations associated with rifampicin and INH resistance in the study population.

In India, MDR TB among new cases are estimated at 2-3% [7]. This study showed low rates (1%) of MDR TB among new sputum positive pulmonary TB patients similar to other Indian studies [9, 10]. The prevalence of INH monoresistance ranging from 1% to 7% has been reported in various Indian studies [11, 12] and our study population showed 1.5% INH monoresistance.

The most common mutation associated with rifampicin resistance was S531L mutation in the* rpoB* region consistent with the findings of various other studies [12–15].

There are publications [16, 17] stating that rifampicin resistance can be a surrogate marker for MDR TB since more than 90% of rifampicin-resistant isolates are also isoniazid resistant. But, a systematic review [18] concluded that rapid tests for rifampicin resistance alone cannot accurately predict rifampicin resistance or MDR TB in areas with low prevalence of rifampicin resistance. However, in areas with high prevalence of rifampicin resistance and MDR TB, these tests may be of value in MDR TB management strategy.

In case of INH resistance, heteroresistant patterns were observed more commonly in our study. Heteroresistance is a preliminary stage towards full resistance which is difficult to detect using conventional DST methods. However, studies on heteroresistance have shown that phenotypic DST results corresponded to the mutated, that is, resistant, organism [19, 20]. The development of heteroresistance has been explained in previous studies as occurring in two ways: the coexistence of two subpopulations (sensitive and resistant) of the same* M. tuberculosis* strain or two separate strains of sensitive and resistant* M. tuberculosis* occurring simultaneously in the same sample. These mechanisms were linked to the clinical entities: new cases, treatment failures, and relapses.

So as explained by Tolani et al. [21], heteroresistance could have arisen due to transmission of both susceptible and resistant bacterial populations from drug resistant patients to previously untreated cases or the presence of exclusively sensitive bacteria at onset which gradually develop resistance during therapy, with incomplete elimination of the sensitive population, and would result in phenotypic resistance but with both forms remaining detectable genotypically.

In this study, the common mutation associated with INH resistance was C15T in the -15 nucleic acid position of the promoter region of* inhA* gene. The prevalence of mutations in the* katG* and* inhA* genes seems to vary widely in different geographic locations as reported in various studies conducted in different countries [12, 22, 23]. In a study by Maurya and et al. [24], the most prominent mutations in* rpoB*,* katG*, and* inhA* genes were S531L, S315T1, and C15T, respectively, similar to this study.

INH resistance occurs due to substitutions in the catalase-peroxidase* katG* gene in more than three-quarters of cases and, more rarely, due to mutation in* inhA* (less than 10%) or the* aphC* gene. The predominant mode of acquisition of resistance via* katG* alteration is the selection of particular mutation, that is, the transversion of codon 315 AGC-ACC (Ser-Thr) that decreases the catalase activity, but maintains a certain level of the peroxidase activity of the enzyme in viable INH-resistant organisms [25].

In this study, the 2 patients with low level INH heteroresistance improved under category I regimen. According to another study [26],* inhA* mutations were not associated with unfavorable treatment outcome. However, the study states that* inhA* mutations do increase the risk of relapse with treatment regimens that contain only INH and ethambutol in the continuation phase as the probability of survival of bacteria with an* inhA* mutation inside macrophages is higher than for the* katG*315 mutant strains because they still have full catalase-peroxidase expression. Whether the increased risk of relapse, in particular for* inhA* mutations, also exists with the World Health Organization- (WHO-) recommended 6-month regimen (2RHEZ/4RH) remains to be studied [26].

The absence of WT8 band in almost 40% of the study population is a matter which needs clarification through further studies. The kit manual mentions that the absence of WT8 band in the* rpoB* region may be attributed to a silent mutation L533P (Leu (CTG) to Pro (CCG)) [6]. One study [27] reported that the change in codon 533 or 515 does not likely correlate with the rifampicin-resistant phenotype. Another study by Raveendran et al. [28] states that such discrepancy may be encountered in this assay and in such cases phenotypic results are considered confirmatory.

However, all the patients in this study with absent WT8 band improved under category I regimen. Furthermore, all these patients were new smear-positive cases with no suspicion of drug resistance. Thus, if deletion of WT8 band is the only indication of rifampicin resistance and there is no mutant probe, it would be advisable to continue the patient on rifampicin therapy till phenotypic sensitivity report is available.

Various studies report different turnaround time for LPA ranging from a period within 24 hours to a period of 5 days [13, 28, 29]. The variation is probably due to lesser frequency of samples being received per day and doing the assay batchwise, only when sufficient samples are present to be done in a single run.

Nonetheless, the use of LPA significantly reduces the turnaround time as compared to solid and automated liquid culture systems. In an average of 3.8 days, the result along with drug sensitivity of rifampicin and INH could be obtained by LPA. Hence, the time to diagnosis and administration of proper treatment is effectively reduced. This has a major impact on reducing the spread of MDR TB and its efficient management.

Different studies have already demonstrated the feasibility of MTBDR*plus* assay as an effective tool in early detection of MDR TB and have good concordance with phenotypic DST. The performance of LPAs has been adequately validated in direct testing of sputum smear-positive specimens and on isolates of* M. tuberculosis* complex grown from smear-negative and smear-positive specimens. However, the direct use of LPA is not recommended on smear-negative-clinical specimens [30].

## 5. Conclusions

The proportion of primary pulmonary MDR TB as well as INH monoresistance was relatively low in the present study group with the most common mutation in the S531L region of* rpoB* gene for rifampicin resistance and C15T in the* inhA* promoter region for INH resistance. This study emphasizes that the implementation of LPA with its rapid turnaround time and high specificity and sensitivity for detection of MDR TB directly from sputum smear-positive samples will be an unprecedented milestone in the diagnosis, treatment, and management of MDR TB. However, the LPA cannot completely replace phenotypic culture methods, as evident by the above results where silent mutations may complicate the clinical interpretation and phenotypic DST is advocated in such cases. A limitation of this study was the inability to perform phenotypic DST due to time and staff constraints.

In conclusion, the LPA definitely aids earlier diagnosis and proper management of MDR TB, although, as with other molecular tests, judicious decision to supplement LPA with conventional methods should be used wherever necessary.

## Figures and Tables

**Table 1:** Category I (DOTS) and category IV (DOTS*plus*) regimen advocated under RNTCP for new TB and MDR TB patients, respectively.

	Intensive phase	Continuation phase
Category I (DOTS)	Rifampicin + INH + ethambutol + pyrazinamide (thrice a week for 2 months)	Rifampicin + INH (thrice a week for 4 months)
Category IV (DOTS*plus*)	Kanamycin + ofloxacin (levofloxacin) + ethionamide + pyrazinamide + ethambutol + cycloserine (daily for 6–9 months)	Ofloxacin (levofloxacin) + ethionamide + ethambutol + cycloserine (daily for 18 months)

**Table 2:** Relationship between diabetes mellitus and sputum smear grade. Higher smear grades were observed in patients who were diabetic. (*P* value: 0.022).

Diabetes	Smear Grade			Total
	1+	2+	3+	
Present	11 (22.4%)	19 (38.8%)	19 (38.8%)	49
Absent	67 (44.4%)	45 (29.8%)	39 (25.8%)	151

Total	78 (39%)	64 (32%)	58 (29%)	200

## References

[1] World Health Organization (2015). *Global Tuberculosis Report 2015*.

[2] Regional Office for South-East Asia (2015). *Tuberculosis in South East Asia Region, Annual TB Report 2015*.

[3] Drug-Resistant TB (2014). Surveillance and response, supplement. *Global TB Report*.

[4] WHO (2008). Molecular line probe assays for rapid screening of patients at risk of MDR-TB. *Policy Statement*.

[5] TB India (2014). *Revised National Tuberculosis Control Programme Annual Status Report*.

[7] Training Module for Medical Practitioners (2010). *Central TB Division, Directorate General of Health Services, Ministry of Health and Family Welfare*.

[8] Jeon C. Y., Murray M. B. (2008). Diabetes mellitus increases the risk of active tuberculosis: a systematic review of 13 observational studies. *PLoS Medicine*.

[9] Sharma S. K., Kaushik G., Jha B. (2011). Prevalence of multidrug-resistant tuberculosis among newly diagnosed cases of sputum-positive pulmonary tuberculosis. *Indian Journal of Medical Research*.

[10] Joseph M. R., Shoby C. T., Amma G. R., Chauhan L. S., Paramasivan C. N. (2007). Surveillance of anti-tuberculosis drug resistance in Ernakulam District, Kerala State, South India. *International Journal of Tuberculosis and Lung Disease*.

[11] D'Souza D. T. B., Mistry N. F., Vira T. S. (2009). High levels of multidrug resistant tuberculosis in new and treatment-failure patients from the revised national tuberculosis control programme in an urban metropolis (Mumbai) in Western India. *BMC Public Health*.

[12] Sharma S., Madan M., Agrawal C., Asthana A. K. (2014). Genotype MTBDR plus assay for molecular detection of rifampicin and isoniazid resistance in *Mycobacterium tuberculosis*. *Indian Journal of Pathology and Microbiology*.

[13] Mohan N., Chandrasekhar P. B., Padmaja I. J., Raizada N., Rao P. S., Kumar B. S. (2014). Genotype MTBDR*plus* line probe assay for rapid and direct detection of rifampicin and isoniazid resistance in *Mycobacterium tuberculosis* complex from sputum samples. *Journal of Dr. NTR University of Health Sciences*.

[14] Hillemann D., Rüsch-Gerdes S., Richter E. (2007). Evaluation of the GenoType MTBDRplus assay for rifampin and isoniazid susceptibility testing of Mycobacterium tuberculosis strains and clinical specimens. *Journal of Clinical Microbiology*.

[15] Lingala M. A. L., Srikantam A., Jain S., Rao K. V. S. M., Ranganadha Rao P. V. (2010). Clinical and geographical profiles of *rpoB* gene mutations in *Mycobacterium tuberculosis* isolates from Hyderabad and Koraput in India. *Journal of Microbiology and Antimicrobials*.

[16] Deepa P., Therese K. L., Madhavan H. N. (2005). Detection and characterization of mutations in rifampicin resistant *Mycobacterium tuberculosis* clinical isolates by DNA sequencing. *Indian Journal of Tuberculosis*.

[17] Drobniewski F. A., Wilson S. M. (1998). The rapid diagnosis of isoniazid and rifampicin resistance in *Mycobacterium tuberculosis*-a molecular story. *Journal of Medical Microbiology*.

[18] Arentz M., Sorensen B., Horne D. J., Walson J. L. (2013). Systematic review of the performance of rapid rifampicin resistance testing for drug-resistant tuberculosis. *PLoS ONE*.

[19] Kumar P., Balooni V., Sharma B. K., Kapil V., Sachdeva K. S., Singh S. (2014). High degree of multi-drug resistance and hetero-resistance in pulmonary TB patients from Punjab state of India. *Tuberculosis*.

[20] Hofmann-Thiel S., Van Ingen J., Feldmann K. (2009). Mechanisms of heteroresistance to isoniazid and rifampin of *Mycobacterium tuberculosis* in Tashkent, Uzbekistan. *European Respiratory Journal*.

[21] Tolani M. P., D'souza D. T. B., Mistry N. F. (2012). Drug resistance mutations and heteroresistance detected using the GenoType MTBDRplus assay and their implication for treatment outcomes in patients from Mumbai, India. *BMC Infectious Diseases*.

[22] Mokrousov I., Narvskaya O., Otten T., Limeschenko E., Steklova L., Vyshnevskiy B. (2002). High prevalence of KatG Ser315Thr substitution among isoniazid-resistant *Mycobacterium tuberculosis* clinical isolates from northwestern Russia, 1996 to 2001. *Antimicrobial Agents and Chemotherapy*.

[23] Van Rie A., Warren R., Mshanga I. (2001). Analysis for a limited number of gene codons can predict drug resistance of *Mycobacterium tuberculosis* in a high-incidence community. *Journal of Clinical Microbiology*.

[24] Maurya A. K., Umrao J., Singh A. K., Kant S., Kushwaha R. A., Dhole T. N. (2013). Evaluation of GenoType*Ⓡ* MTBDR*plus* assay for rapid detection of drug susceptibility testing of multi-drug resistance tuberculosis in Northern India. *Indian Journal of Pathology and Microbiology*.

[25] Veluchamy M., Madhavan R., Narayanan S., Rajesh L. (2013). KatG gene as a surrogate molecular marker leading to cause drug resistance in *Mycobacterium tuberculosis* isolates. *American Journal of Infectious Diseases and Microbiology*.

[26] Huyen M. N. T., Cobelens F. G. J., Buu T. N. (2013). Epidemiology of isoniazid resistance mutations and their effect on tuberculosis treatment outcomes. *Antimicrobial Agents and Chemotherapy*.

[27] Ohno H., Koga H., Kohno S., Tashiro T., Hara K. (1996). Relationship between rifampin MICs for and *rpoB* mutations of *Mycobacterium tuberculosis* strains isolated in Japan. *Antimicrobial Agents and Chemotherapy*.

[28] Raveendran R., Wattal C., Oberoi J. K., Goel N., Datta S., Prasad K. J. (2012). Utility of GenoType MTBDR*plus* assay in rapid diagnosis of multidrug resistant tuberculosis at a tertiary care centre in India. *Indian Journal of Medical Microbiology*.

[29] Anek-vorapong R., Sinthuwattanawibool C., Podewils L. J. (2010). Validation of the GenoType® MTBDRplus assay for detection of MDR-TB in a public health laboratory in Thailand. *BMC Infectious Diseases*.

[30] Patra S. K., Jain A. (2012). Molecular diagnosis of multi drug resistant tuberculosis. *International Journal of Biomedical and Advance Research*.

